# Application of nanomaterials in diagnosis and treatment of glioblastoma

**DOI:** 10.3389/fchem.2022.1063152

**Published:** 2022-12-09

**Authors:** Shuangqi Yu, Lijie Chen, Hongyu Xu, Shengrong Long, Jiazhi Jiang, Wei Wei, Xing Niu, Xiang Li

**Affiliations:** ^1^ Department of Neurosurgery, Zhongnan Hospital, Wuhan University, Wuhan, Hubei, China; ^2^ Brain Research Center, Zhongnan Hospital, Wuhan University, Wuhan, Hubei, China; ^3^ China Medical University, Shenyang, Liaoning, China

**Keywords:** glioblastoma, glioma, tumor targeting, nanomaterial, blood-brain barrier

## Abstract

Diagnosing and treating glioblastoma patients is currently hindered by several obstacles, such as tumor heterogeneity, the blood-brain barrier, tumor complexity, drug efflux pumps, and tumor immune escape mechanisms. Combining multiple methods can increase benefits against these challenges. For example, nanomaterials can improve the curative effect of glioblastoma treatments, and the synergistic combination of different drugs can markedly reduce their side effects. In this review, we discuss the progression and main issues regarding glioblastoma diagnosis and treatment, the classification of nanomaterials, and the delivery mechanisms of nanomedicines. We also examine tumor targeting and promising nano-diagnosis or treatment principles based on nanomedicine. We also summarize the progress made on the advanced application of combined nanomaterial-based diagnosis and treatment tools and discuss their clinical prospects. This review aims to provide a better understanding of nano-drug combinations, nano-diagnosis, and treatment options for glioblastoma, as well as insights for developing new tools.

## 1 Introduction

Glioblastoma (GBM) is a general term for tumors derived from glial cells and neuronal cells. It is the most common malignant tumor in the brain, accounting for 40%–50% of all intracranial tumors (Chen et al., 2017). The World Health Organization (WHO) classifies gliomas by cell type: astrocytoma, glioblastoma, and oligodendroglioma, and by malignancy grade (WHO I, II, III, IV). In 2021, the WHO CNS5 updated the molecular biomarkers for different tumor types, bringing more benefits and meaningful guidance to clinical practice. The WHO CNS5 has taken a new approach to classifying gliomas, glioneuronal tumors, and neuronal tumors, dividing them into six families: adult-type diffuse gliomas, pediatric-type diffuse low-grade gliomas, pediatric-type diffuse high-grade gliomas, circumscribed astrocytic gliomas, glioneuronal and neuronal tumors, and ependymomas (Louis et al., 2021). At present, temozolomide chemotherapy combined with radiotherapy after maximum feasible resection is the primary clinical treatment for adult primary GBM. With the emergence of many targeted therapies, bevacizumab and programmed death -1 (PD-1) have also been used in the standardized treatment of GBM (Khasraw et al., 2012; Cloughesy et al., 2019; Zhao M. et al., 2020; Detti et al., 2021). However, GBMs grow infiltratively and are not clearly demarcated from normal tissues. They are, therefore, difficult to completely remove surgically and have high recurrence rates. Meanwhile, the blood-brain barrier (BBB), a highly selective semipermeable structural and chemical barrier (Cai et al., 2018), hinders drug delivery to the brain. Thus, drugs rarely reach effective therapeutic concentrations at the tumor site, limiting the effect of radiotherapy and chemotherapy. Due to these obstacles, GBMs have high mortality rates. To overcome this, nanomaterials have been used as a new treatment modality. They have many interesting characteristics, such as small size and targetable transport, making them good delivery tools for drugs, genes, or proteins across cells or the BBB.

Nanomaterials are materials with at least one dimension in the three-dimensional space in the nanoscale or composed of such materials as basic units. Nanomaterials have small particle sizes, controllable texture, and strong plasticity. In GBM treatment, nanomaterials are mainly used as carriers for radiotherapy and chemotherapy drugs (Zhao M. et al., 2020), helping drugs cross the BBB and maintain the necessary blood drug concentration. Additionally, nanomaterials can cause tumor cell necrosis by affecting the tumor microenvironment (Zanganeh et al., 2019). For example, a nanocomposite drug consisting of a polyglycerol functionalized doxorubicin-containing nanodiamond was designed to re-adjust the inhibitory glioblastoma immune microenvironment. This nanocomposite provided GBM with immunosuppressive microenvironment by activating autophagy. This rebuilding promoted the anti-GBM immune response and strengthened the activation of dendritic cells (Li et al., 2019).

The primary purpose of nanomaterials is to improve the solubility, stability, and effective concentration of drugs and reduce their systemic toxicity (Zhao M. et al., 2020). The main nanomaterials used in current research are nanoparticles (NPs). They can be inorganic, polymeric, or bionic. Each type has its advantages. Inorganic NPs, for example, are highly stable, and their physical and chemical properties can be adjusted by using suitable materials and sizes (Bharti et al., 2019). In addition, polymeric nanoparticles can encapsulate drugs by electrostatic or covalent bonding, maintaining their blood concentration and thereby improving the bioavailability of drugs. Most importantly, modified polymeric NPs can aggregate at specific sites. Biomimetic nanocomposites have higher biological stability and can escape the immune system, allowing drugs to act on the target continuously without being cleared by the liver and kidney. Besides nanoparticles, other nanomaterials (e.g., liposomes, quantum dots, cellular and extracellular vesicles, or virus-like particles) are frequently used in GBM treatment research.

The emergence of nanomaterials has brought opportunities for the diagnosis and treatment of GBM, and their various characteristics can help overcome the current clinical challenges of GBM. Here, we review the current progress on the applications of nanomaterials in GBM treatment research to provide insights for developing new nano-drug combinations, nano-diagnosis, and GBM treatment schemes.

## 2 Blood brain barrier and delivery mechanism of nanomaterials

### 2.1 Blood brain barrier

The blood-brain barrier (BBB) is a specialized structure within the central nervous system that acts as a physical and metabolic barrier restricting transport between the blood and neural tissues. It consists of brain microvascular endothelial cells, pericytes, astrocytes, neurons, and a basement membrane. This physical barrier protects the brain and maintains the stability of the intracranial environment. The excellent barrier properties of the BBB protect the brain from harmful macromolecules and pathogens present in the blood (Xie et al., 2019). However, this barrier also hinders traditional drug delivery and affects drug efflux. Currently, most small-molecule drugs and almost all macromolecular drugs (e.g., recombinant proteins, therapeutic antibodies, and nucleic acids) cannot cross the BBB (Baratta, 2018). Thus, more and more researchers pay attention to nanomaterials, as shown in many clinical trials on the diagnosis and treatment of GBM (Dong, 2018). The delivery mechanisms of nanomaterials can be divided into passive targeting and active targeting (Zhao M. et al., 2020).

### 2.2 Passive targeting

In passive targeting, a drug of a specific size is injected through the BBB into the abnormal, porous vascular endothelium of the tumor. Since the tumor vascular endothelium lacks appropriate drainage, the drug remains in the tumor area for a long time. This phenomenon is also known as the enhanced permeability and retention (EPR) effect and was first proposed by Matsumura and Maeda in 1986 (Matsumura and Maeda, 1986). However, current studies have shown that the EPR effect is unstable and varies significantly among different tumors. Besides, the EPR effect achieved in rodent models cannot be reproduced clinically because human tumors have heterogeneity or lack fenestrations in the tumor endothelium, acidic and anoxic areas, low and heterogeneous pericyte coverage, and high interstitial fluid pressure induced by a dense extracellular matrix (Danhier, 2016).

### 2.3 Active targeting

Active targeting is a non-invasive approach that involves transporting drugs to target organs using site-specific ligands. In particular, drug-loaded nanocarriers that can target brain capillary endothelial cells and brain tumor cells show potential in oncology (Béduneau et al., 2007). In active targeting, nanocarriers enter cells by taking advantage of ligand-receptor interactions. So far, active targeting nanomaterials have been applied to the diagnosis and treatment of various malignant tumors, such as liver cancer, lung cancer, and lymphoma (Falgàs et al., 2020; Kaps and Schuppan, 2020). Developing active targeting nanomaterials able to cross the BBB requires understanding how to use the brain capillary endothelium. Nevertheless, the active targeting of the BBB represents a promising non-invasive strategy for improving anti-glioblastoma drug delivery (Miranda et al., 2017). Active targeting can be further subdivided into adsorptive-mediated, carrier-mediated, receptor-mediated, and cell-mediated delivery, which target cells in different ways.

#### 2.3.1 Adsorptive-mediated delivery

Adsorptive-mediated endocytosis (AMT) can deliver drugs to the brain through the BBB by allowing cationic molecules to bind to and be adsorbed onto the surface of the endothelial cell lumen. Adsorption-mediated transcytosis is initiated by the electrostatic interaction between positively charged ligands and negatively charged cell membranes. Nanocarriers then enter cells through clathrin-dependent endocytosis (Patel and Patel, 2017). Cationic proteins combined with cell-penetrating peptides (CPPs) can improve transport. With their short amino acid sequence, CPPs can interact with cell membranes and pass through cell membranes by energy-dependent and energy-independent mechanisms. Studies have shown that cationic CPPs have significantly more flux to the brain parenchyma than amphiphilic CPPs *in vivo* (Komin et al., 2017). Due to electrostatic interactions, cationic CPPs are easily captured by intracellular organelles to some extent. The drug-CPP linkage type substantially affects their ability to cross the BBB. Liu et al. (2014) compared the effects of amide, maleimide, and disulfide linkers linking the endorphin 1 to the CPP synB3 on BBB crossing efficiency. The disulfide linkage was the most efficient, and it was able to release the free drug in the brain. The adsorption-mediated transcytosis combined with a targeting strategy can effectively improve the EPR effect and reduce nonspecific uptake. Srimanee et al. (2018) developed a non-covalent CPP-targeting peptide (CPP-TP) complex with the CPP PepFect 14 and a hexaglutamate-modified angiopep-2 (ANG), as a targeting peptide. This complex showed enhanced penetration ability and glioblastoma cell specificity as an siRNA carrier. During the last decades, nanoparticles with various compositions have been developed, such as polymeric nanoparticles (PPs), gold NPs, gadolinium NPs, selenium NPs, or protein-based NPs (Gupta and Sharma, 2019).

#### 2.3.2 Carrier-mediated delivery

Carrier-mediated delivery is initiated by combining a designed nanocarrier and a specific transporter protein (Bourganis et al., 2018). This drug delivery system consists of nanocarriers (such as liposomes, NPs, polymeric micelles, dendrimers, or polymersomes) and ligands for various receptors, including transferrin receptor (TfR), lactoferrin receptor (LfR), low-density lipoprotein receptor (LDLR), and folate receptor (FR) (Chen et al., 2014; Wang et al., 2015). Ligand-modified drug carriers deliver drugs to the receptor-containing target cells like “guided missiles” (Liu et al., 2021).

Based on transport direction and substrate, transport modes can be divided into three categories: 1) The system pumping blood into the brain, which transports essential nutrients to the brain, including glucose, amino acids, and nucleotides. 2) The drug efflux pumps expelling exogenous substances out of the peripheral circulation to prevent them from entering the brain. 3) The efflux system from the brain to the blood, which mainly removes metabolic waste and neurotoxic substances in the interstitial fluid of the brain. Among them, the pumping system is the breakthrough point of carrier-mediated delivery. Nanocarriers going through the pumping system are generally designed as nutritional analogs with a high affinity for transporters, so their molecular weight is generally small. Cell-mediated delivery can be used in anticancer therapy (Naik et al., 2021). For example, Naik et al. (2021) developed doxorubicin-containing liposomes and confirmed that conjugating these liposomes with a ligand mimic increased their antiproliferative activity on cancer cells overexpressing the corresponding receptor.

#### 2.3.3 Receptor-mediated delivery

Targeting receptors that mediate endocytosis allows more robust targeting than with adsorption-mediated and carrier-mediated delivery because of the high specificity of the ligand-receptor interaction. In one study, cationic liposomes loaded with temozolomide were encapsulated in a multilayer crown of plasma proteins with a natural affinity for the folic acid (FA) receptor, which is highly expressed in the BBB (Tang et al., 2021). In an *in vitro* BBB model, these cationic liposomes with multilayered biomolecular crowns exhibited high ingestion by endothelial cells of human umbilical vein, which promoted the anticancer effect of temozolomide in U-87 MG cells (Arcella et al., 2018). Mram Alho *et al.* (Ramalho et al., 2018) developed stable polylactic acid-co-glycolic acid nanoparticles functionalized with the OX26 monoclonal antibody for the transferrin receptor. These nanoparticles delivered temozolomide, an anti-glioma agent.

Up to now, researchers have used many ligands of receptors on the BBB or glioma cells as targeting moieties for BBB crossover and/or glioma-targeted drug delivery, such as peptides (Wang et al., 2015). In addition, more and more studies have proved that BBB/glioma-specific targeting nanocarriers can help drugs selectively target glioma cells, increasing their therapeutic efficiency while reducing systemic toxicity (Wang et al., 2017; Ramalho et al., 2018). Receptor-mediated drug delivery may allow membrane-impermeable drugs to penetrate target cells and activate natural signaling cascades (McPherson et al., 2001).

Besides directly targeting tumor surface receptors, the receptor-mediated pathway includes three important steps, including the formation of ligand-receptor complexes, transport through the cytoplasm of endothelial cells, and extracellular secretion outside the base of the BBB (Azarmi et al., 2020). In the second step, the lysosomal system may threaten the integrity of the drug. This can be bypassed by using cationic molecules and pH-sensitive drug carriers (Shir et al., 2006). However, receptor-mediated transfer also faces some problems. The high affinity leads to strong interactions between multiligand receptors on the lumen side of the BBB, limiting the entry of therapeutic molecules into the brain parenchyma. By contrast, using ligands with lower affinities can lead to higher drug release into the brain, but this requires administering higher doses, which is usually not applicable (Clark and Davis, 2015).

#### 2.3.4 Cell-mediated delivery

**FIGURE 1:**
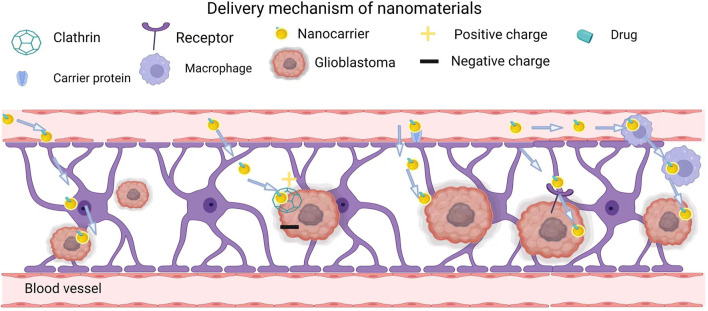
Delivery mechanism of nanomaterials.

In recent years, cell-mediated transcytosis has received increasing attention because immunogenicity and instability can hinder the use of antibodies and peptides (Yu et al., 2016). Neural stem cells, mesenchymal stem cells, neutrophils, macrophages, and exosomes, among others, have an intrinsic tumor-homing ability, allowing them to target malignant GBMs for drug delivery (Cho et al., 2019). Neutrophils have been widely studied in the treatment of brain tumors, especially for the treatment of postoperative recurrent tumors. For example, inspired by the ability of macrophages to cross the BBB, Tingting *et al.* encapsulated catalase into silica nanoparticles to produce a nanoplatform called CAT@SiO_2_-ICG (CSI). Next, they further encapsulated CSI into AS1411 aptamer-modified macrophage exosomes to form CSI@Ex-A (Wu et al., 2022) (Figure 1). Similarly, recent studies have confirmed that cell-mediated delivery systems can contribute to the clinical treatment of gliomas. Xue et al. (2017) have shown that neutrophils carrying liposomes that contain paclitaxel, can penetrate the brain and inhibit GBM recurrence in mice whose tumors have been surgically removed. Similarly, one study demonstrated that the dendritic cell-mediated delivery of doxorubicin-polyglycerol-nanodiamond composites stimulated GBM cells immunogenicity and elicited an anti-glioblastoma immune response (Li et al., 2018). These researches have revealed the feasibility of cell-mediated delivery for GBM treatments and laid the foundation for a translational study of this therapeutic paradigm to improve clinical outcomes in patients with malignant brain tumors.

### 2.4 Nano-assisted GBM diagnosis

Besides treating tumors, nanomaterials can be used in combination with imaging modalities, such as computed tomography (CT), functional magnetic resonance imaging (FMRI) (Richiardi et al., 2011), and positron emission tomography (PET) (Fink et al., 2015) to increase the sensitivity and accuracy of tumor detection. Moreover, ultrasound (Imbault et al., 2017) and fluorescence imaging (Li et al., 2017) have been widely used in the clinical diagnosis and treatment of GBM. First, under normal physiological conditions, the physical and chemical barriers of BBB can efficiently transport necessary particles to human brain and selectively discharge harmful or excessive materials (Wu et al., 2019). Under pathological situations, practically all kinds of macromolecular drugs (proteins, antibodies, peptides, developers, etc.,) and small molecular drugs can pass through the BBB (Umlauf and Shusta, 2019). Although multiple brain tumor diagnosis strategies exist, their various limitations have affected their efficacy in the diagnosis of GBM. Second, the perpetual drainage and circulation of blood-cerebrospinal fluid and interstitial fluid stop most macromolecules from entering the bloodstream and diagnostic drugs from entering the brain. Moreover, tumor-acquired characteristics of the brain prevent drug from penetrating into tumors. In particular, tumor-induced endothelial cell tight junction damage increases drug penetration, leading to a heterogeneous distribution (Groothuis, 2000; Roose et al., 2003; Arvanitis et al., 2020).

Magnevist, an extensively used clinical contrast agent, plays an important role in evaluating tumors and their recurrence in magnetic resonance imaging (MRI) (McDannold et al., 2012; Kim, 2020). However, due to its short half-life, maintaining a sufficient concentration in the tumor requires repeated high-dose injections. Vascular leakage (Zhao H. L. et al., 2020), pseudoprogression (Brandsma et al., 2008), and pseudoreaction (Batchelor et al., 2007; Dhermain et al., 2010) after radiotherapy or anti-angiogenesis therapy also affect MRI accuracy. Besides, magnetic resonance spectroscopy and PET are often used to quantify and describe the development of cerebral tumors. Integrated with tracers and positron radionuclides in existence, they will achieve evaluable and reliable tissue autoradiography (Kratochwil et al., 2019), providing important information needed in diagnosis on tumors. But it is the requirements for accurate diagnosis of cerebral tumors that still cannot meet. In response to these hurdles (substandard specificity and accuracy, ephemeral contrast agent half-life, large requirement for imaging, etc.,), and to improve imaging sensitivity in diagnosis, nanoparticles have been developed.

#### 2.4.1 Nanomaterials for MRI

The properties of nanomaterials greatly help overcome the difficulties faced by traditional radiographic agents. For example, Self-assembled nanoparticles of amphiphilic gadolinium chelates show extremely high Gd3 + loading capacity for enhanced imaging (Othman et al., 2011). Coupling gadolinium to interleukin 13 (Li et al., 2015), an arginine-glycine-aspartate (RGD) peptide (Zhan et al., 2010; Sun et al., 2014; Richard et al., 2017), an epidermal growth factor receptor (EGFR) mutant antibody (EGFRvIII) (Hadjipanayis et al., 2010) or anti-gd2 antibody (Shah et al., 2013) can markedly enhance tumor targeting. Gadolinium metal fullerene nanoparticles have a cage surface charged by amino (-NH^3+^), showing incredible 1H MR relaxation. These data suggest that composite nanoprobes can serve as alternatives to magnetic resonance contrast agents (Lajous et al., 2018). The clinical applications of nanodiagnostic agents consisting of other magnetic materials especially iron and manganese, have also been researched. Superparamagnetic iron oxide nanoparticles (SPIO) have been paid tremendous clinical attention due to their magnetic properties. For example, the tungsten-doped iron oxide crystal (WFe) contrast agent has a sterling T1-weighted effect. The WFe nanoparticles possessed high average T1, 22% shorter than that of ferritin at the injection site (Clavijo Jordan et al., 2014). In addition, the superparamagnetism of SPIO is beneficial to the aggregation of the magnetic target. For example, the synergistic release of SPIO by focused ultrasound and magnetic targeting notably increases its accumulation in the brain parenchyma (Lee et al., 2019). In addition, modifying SPIO with a brain tumor-targeting peptide cRGD (Richard et al., 2017), a targeting antibody EGFRvIII (Hadjipanayis et al., 2010), or the targeting toxin chlorotoxin (Stephen et al., 2014) can enhance its tumor-targeting specificity. Finally, manganese, chelated with albumin-binding molecules, has been evaluated as a new contrast agent in both subcutaneous and *in situ* brain tumor models (Zhou et al., 2019).

#### 2.4.2 Nanomaterials for CT imaging and Surface Enhanced Resonance Raman Scattering

Gold nanoparticles have incredible biosafety properties and are readily synthesized. Thus, a series of gold NPs with Surface Enhanced Resonance Raman Scattering (SERRS) signals were developed to guide brain tumor resection (Saha et al., 2012; Karabeber et al., 2014). These nanoparticles show strong signal intensity in SERRS after 24 h of injection, yielding a clear tumor contour. Besides gold nanoparticles, a new PET/CT imaging reagent named ^68^galliumBNOTA-PRGD2 (68 Ga-PRGD2) showed some GBM diagnosis success (Li et al., 2014). Since GBM cells overexpress avb3, the target integrin of 68 Ga-PRGD2, the nanoparticle accumulates in GBM cells. The sensitivity and specificity of 68 Ga-PRGD2 for GBM grading were respectively 12% and 25% superior to those of the clinical PET/CT agent 18F-fluorodeoxyglucose (FDG). Moreover, Zhao et al. (2016) used the integrin a5b1 to enhance the specificity of 99mTc-HisoDGR SPECT/CT probes. 99mTcHisoDGR yielded precise contours in subcutaneous and *in situ* models 1.5 and 2 h after injection, respectively.

#### 2.4.3 Nanomaterials for optical imaging

Currently, fluorescence imaging surgery guidance is widely used in clinical practice. Nanoparticles can be used as fluorescent dye carriers or fluorescent dyes to enhance optical imaging, dramatically reducing the failure rate of surgery (Wu et al., 2019). Several nanoparticles, including liposomes and polymer NPs, can effectively deliver live imaging fluorescent dyes to brain tumor sites during the operation. Indocyanine green, a near-infrared fluorescent probe approved by the FDA, is widely used in tumor tracking and photothermal therapy. Liposome nanoparticles (LP-iDOPE) combined with VEGF-bevacizumab can enhance brain tumor imaging. One day after injection and before surgery, LP-iDOPE enhanced tumor localization (Suganami et al., 2015). Biodegradable polymers, such as polyalkylcyanoacrylate (Vauthier et al., 2003) and poly (lactic-co-glycolic acid) (Han et al., 2016; Guan et al., 2017; Orunoğlu et al., 2017), are widely used to prolong the retention period of nanoparticles *in vivo*. Besides, polymer nanoparticles can load cerebral tumor targeting particles like chlorotoxin or anti-PDGFRD antibody (Monaco et al., 2017). A novel optical contrast agent with good biocompatibility was developed by modifying a polymer nanomatrix with fluorescent dyes and silver nanosheet clusters. The contrast of these nanoparticles was 90% higher than that of the control (Ray et al., 2014).

Being activated by light at a particular wavelength, some nanoparticles can emit light at another (such as quantum dots (Liu M. X. et al., 2017; Tang et al., 2017) and upconversion luminescent materials (Wang et al., 2020)), making them ideal fluorescent dyes for cancer targeting and imaging. Quantum dots are widely used for biological imaging diagnosis due to their excellent water solubility, low fluorescence quenching rate, high fluorescence quantum yield, and stable chemical properties (Wu and Yan, 2013). Thus, quantum dots modified with tumor-targeting molecules can achieve specific biological imaging. Moreover, near-infrared technology can increase their fluorescence and Raman signals by several orders of magnitude (Gill and Le Ru, 2011), making them more sensitive for imaging in preclinical and clinical studies. Upconversion nanoparticles have considerable light stability, no fluorescence scintillation, deep tissue penetration, and low light damage effects (Gu et al., 2013; Liu et al., 2021; Mohan and Poddar, 2021), making them promising *in vivo* imaging probes (Idris et al., 2012; Yang et al., 2012). However, although these nanoparticles have strong tissue penetration ability, they cannot penetrate brain tissue like traditional fluorescent dyes (Li and Wang, 2018). To overcome this problem, research on second near-infrared transparent window (NIR-II, 1,000–1700 nm) fluorescent nanoprobes has gradually developed. NIR-II fluorescent nanoprobes have a stronger ability to penetrate tissues and achieve higher image fidelity. Ag2S quantum dots are one of the typical NIR-II fluorescent probes. Coupled to cyclic RGD peptides, the biocompatible NIR-II Ag2S fluorescent probe can achieve targeted labeling and imaging of U87 cells (Zhang et al., 2012). Considering the enhanced penetration depth of fluorescence signals, Qi et al. (2018) further analyzed the tissue penetration depth of NIR-II imaging probes. Near-infrared aggregation-induced emission under excitation with a 1,300 nm NIR-ii laser allowed the group to observe 5 mm blood vessels at a depth of 1,065 mm in the brain.

#### 2.4.4 Nanomaterials for multimodal imaging

Since each diagnostic method has its own advantages and disadvantages, combining different diagnostic methods can optimize the outcomes. The development of dual-mode imaging nanoparticles has majorly impacted biomedical research. Angiopep-2 (ANG, TFFYGGSRGKRNNFKTEEY) coupled with upconversion dual-mode imaging nanoparticles (ANG/PEG-UCNPs) and Gd were constructed for targeting GBM. Compared with non-ANG and Gd-DTPA imaging, the nanoprobe yielded a significantly enhanced T1-weighted magnetic resonance contrast for glioblastoma. T2-weighted MRI also shows great potential for identifying clear glioblastoma borders (Ni et al., 2014). These results confirmed the advantage of MRI combined with fluorescent nanoprobes in GBM diagnosis. However, although dual-mode imaging improves the accuracy of high-resolution information, it still does not achieve overall tumor visualization. To improve the diagnosis accuracy and sensitivity, various imaging nanoparticles have been explored. For example, gold silicon-based SERS nanoparticles were used for three-mode imaging applications, where gold NPs were encapsulated by Gd^3+^ ions. MRI, photoacoustic imaging, and SERS showed clear tumors. The three-dimensional rendering of the magnetic resonance and photoacoustic images showed good co-expression signals in the tumor (Kircher et al., 2012; Neuschmelting et al., 2018).

#### 2.4.5 Summary

Traditional GBM diagnosis faces some tough obstacles, such as the BBB and tumor heterogeneity. Although nanotechnologies have solved these problems to a certain extent, single-dimension diagnosis remains extremely limited due to the complexity of GBM. Combining multiple diagnostic methods will become a critical research field. Therefore, it is essential to construct various nanomaterials meeting the requirements for multi-mode combinations. Table 1 lists existing diagnosis nanomaterials and their related mechanisms.

**TABLE 1:** Nanomaterialbased diagnostic for glioma.

Application	Nanomaterial	Highlight
MR imaging	Self-assembling nanoparticles of amphiphilic gadolinium chelates	High loading capacity of Gd3+ ions, enhanced imaging effect
	conjunction of gadolinium with interleukin (IL)-13, the arginine–	Significantly enhanced tumor targeting ability
	Glycine–aspartic acid (RGD) peptide, the epidermal growth factor receptor (EGFR) deletion mutant (EGFRvIII) antibody or the anti-GD2 antibody	
	Gadolinium metallofullerene nanoparticles	Excellent 1H MR relaxivity
	Tungsten doped iron oxide crystal	Excellent T1-weighted effect
	Superparamagnetic iron oxide nanoparticles	Magnetically controlled target accumulation
	SPIO with brain tumor targeted peptides cRGD, targeted antibody EGFRvIII and targeted toxin chlorotoxin (CTX)	Specific targeting in tumors
Surface Enhanced Resonance Raman Scattering (SERRS) and CT imaging	Au NPs	A stronger SERRS signal intensity 24 h postinjection, and then an accurate outline of the tumor
	68 Ga-PRGD2	Enhanced sensitivity and specificity of glioma grading
	99 mTc-HisoDGR SPECT/CT	Clear visualization in both subcutaneous and orthotopic models respectively 1.5 h and 2 h post injection
Optical imaging	Indocyanine green (ICG) loaded liposomal formulated nanoparticles (LP-iDOPE)	Enhanced imaging effect of brain tumors, excellent tumor-specific localization
	Poly-alkyl-cyano acrylates (PACA) and poly lacticcoglycolic acid (PLGA)	Extending the circulation time of nanoparticles in the body
	Polymer nano matrix loaded with silver nanoplate clusters and a fluorescent dye	Better biocompatibility and contrast
Multimodal imaging	Quantum dots (QDs)	Good solubility in water, high fluorescence quantum yield, low fluorescence quenching rate and stable chemical properties
	Quantum dots modified with tumor targeting molecules	Specific bioimaging
	Upconversion nanoparticles (UCNPs)	Good photostability, no fluorescence scintillation, deep tissue penetration and small photo damage
	Ag_2_S QDs	Deeper penetration potential through tissues, higher fidelity of images
	Angiopep-2 (ANG) dual-targeting simultaneously Gd-doped upconversion dual-mode imaging nanoparticles (ANG/PEG-UCNPs)	Enhanced T1-weighted MR contrast of glioblastoma, great potential in T2-weighted MRI, ability to show a clear glioblastoma boundary
	Gold–silica-based SERS nanoparticles	Ability to show clear tumor visualization by three modalities in triple-modality imaging

### 2.5 Nano-assisted GBM therapy

#### 2.5.1 Ferroptosis

Ferroptosis is an iron-dependent programmed cell death distinct from apoptosis, necrosis, pyroptosis, and autophagy (Bogdan et al., 2016; Zheng et al., 2017; Shen et al., 2018; Zhang et al., 2020). Excessive iron reacts with hydrogen peroxide (H2O2), generating hydroxyl radicals and singlet oxygen in cells (this process is known as the Fenton reaction). High hydroxyl radical levels eventually lead to cytotoxic lipid peroxidation. Since ferroptosis and apoptosis are radically different in mechanism and phenotype, combination therapy targeting these two processes may be a strategy for treating GBM. Based on this idea, Yulin *et al.* proposed an innovative local chemotherapy approach. They constructed iron oxide nanoparticles (IONPs) based on gene therapy to treat patients with glioblastoma via ferroptosis and apoptosis after surgery. By modifying the porous structure of carboxyl-linked IONPs, they co-transferred small interfering RNA (siGPX4, targeting glutathione peroxidase 4) and cisplatin with a high drug loading efficiency. During intracellular degradation, IONPs markedly increased iron (Fe^2+^ and Fe^3+^) levels and activated reductive nicotinamide adenine dinucleotide phosphate (NADPH) oxidase (NOX), increasing H_2_O_2_ levels. The Fenton reaction between Fe^2+^, Fe^3+^, and intracellular H_2_O_2_ produced reactive oxygen species and initiated ferroptosis, while cisplatin destroyed nuclear and mitochondrial DNA, leading to apoptosis. Simultaneously, si-GPX4 was released, inhibiting GPX4 expression and produced a synergistic effect through mechanisms related to ferroptosis. Therefore, this system achieved an excellent therapeutic effect and low systemic toxicity both *in vitro* and *in vivo* (Zhang et al., 2020).

#### 2.5.2 Gene therapy

Gene therapy is a potential method for the treatment of GBM. In this context, nanomaterials are mainly used as carriers for genes designed to treat tumors. The designed genes can be suicide genes (that convert nontoxic prodrugs into cytotoxic drugs), immunoregulatory genes (that stimulate the immune system), or tumor suppressor genes (Dixit and Kumthekar, 2017). In addition, Qiang *et al.* constructed lipid-polymer hybrid nanoparticles (LPHNs-cRGD) to efficiently and specifically deliver a CRISPR/Cas9 plasmid targeting the temozolomide resistance gene O-6-methylguanine-DNA methyltransferase (MGMT). To facilitate the entry of the genes into the GBM *in vivo*, they non-invasively and locally gained access into the BBB using focused ultrasound microbubbles. The nanocarrier successfully mediated the transfection of pCas9/MGMT, downregulating MGMT expression and increasing the sensitivity of GBM cells to temozolomide (Yang et al., 2021).

#### 2.5.3 Radiotherapy

Radiotherapy is a common treatment method for malignant tumors. However, radiation damages normal tissues and tumor hypoxia can lead to radiation resistance. There are two solutions to overcome these problems: the first is to use more advanced radiotherapy technology, and the second is to develop a new generation of therapeutic agents able to sensitize tumor cells to ionizing radiations to improve their effect. Nanomaterials can be used as radiosensitizers or their carriers. They have achieved good results as radiosensitizers or carriers after photon and particle radiation (Caban-Toktas et al., 2020; Chung et al., 2020; Kazmi et al., 2020; Ruiz-Garcia et al., 2021). Some nanoparticles, such as gadolinium, gold, hafnium, bismuth, and platinum nanoparticles, can also achieve good results as sensitizers (Lux et al., 2019). The principle is that when photons and particles activate the nanoparticles, a photoelectric effect (Kazmi et al., 2020) amplifies the radiation effect (Lacombe et al., 2017; Kuncic and Lacombe, 2018). Gold nanoparticles became an important radiosensitizer due to their biocompatibility, tunable optical properties, and high stability. Kunoh et al. (2019) compared the radiosensitivity of gold nanoparticle-treated and untreated cells. Their experiments showed that pre-treating cells with gold NPs prevented radioresistance development in cancer cells. Interestingly, the gold NPs did not induce apoptosis but increased the number of abnormal nuclei, causing mitotic cell catastrophe.

#### 2.5.4 Photothermal therapy

Photothermal therapy consists in injecting materials with high photothermal conversion efficiency into the body, making them accumulate around the tumor by targeted recognition technology, then irradiating them with an external light source (generally infrared light) to convert light into heat and kill cancer cells. Based on the above ferroptosis treatment, Yulin *et al.* blended gallic acid with Fe^2+^ to form gallic acid/Fe^2+^ nanoparticles with excellent photothermal conversion ability. Near-infrared light irradiation (808 nm) can drastically improve the Fe^2+^ release efficiency of nanoparticles and induce ferroptosis in tumor cells while releasing a large amount of heat to kill tumor cells (Zhang et al., 2021).

#### 2.5.5 Magnetothermal therapy

Magnetothermal therapy-mediated cancer therapy (MHCT) consists in exposing magnetic nanomaterials to an alternating magnetic field to heat tumor tissue and alter cellular mechanisms. A temperature rise from 37°C to 42°C–45°C can induce tumor cell death by activating specific intracellular and extracellular degradation mechanisms (Krawczyk et al., 2011; Zhang and Calderwood, 2011; Gupta and Sharma, 2019). At 42°C, tumor cells undergo irreversible damage leading to apoptosis, while achieving the same effect in normal cells requires at least 55°C.

However, magnetothermal therapy has not yet become one of the main GBM therapy because some challenges remain. First, the safety, efficacy, and appropriate dose range of MHCT are unclear, and this needs to be taken into account to determine the magnetic nanomaterial dose. Moreover, the choice of magnetic parameters and the appropriate magnetic field strength are also undetermined. Second, injecting effective drugs into targeted GBM cells through clinically feasible methods remains challenging. Most small-molecule drugs cannot penetrate the BBB, which dramatically hinders the delivery of drugs to tumor sites. In addition, some physical limitations affect hyperthermia performance. These include heat distribution, toxicity, magnetic nanosensors efficiency, and the reduction of hyperthermia performance of magnetic nanoparticles (MNPs) in the cellular environment, that is, once they are internalized by the cell (lysosomal aggregation phenomenon) (Di Corato et al., 2014; Soukup et al., 2015). Besides, the lack of methods to accurately measure local body temperature is another obstacle to MNP treatment evaluation (Dewhirst et al., 1997; Arthur et al., 2005). More importantly, achieving the precise targeting of tumor cells by MNPs is also one of the main challenges for GBM treatment.

According to a recent meta-analysis, less than 1% of injected particles accumulate at the tumor site. Thus, the use of targeted strategies to attach specific targeted moieties to the surface of nanomaterials has also become an important unsolved question. However, in some techniques, only 4% of the targeted portion of the used ligand is recognized by its targeted receptor, which may lead to heterogeneity and poor results (Herda et al., 2017). In addition, converting targeted strategies from basic research to clinical research is ineffective, especially MHCT. A study reported that in mouse xenograft models, the accumulation of antibodies usually varies between 0.5% and 50% of the injection dose per Gram of tumor tissue. Meanwhile, we observed that the accumulation of antibodies per Gram of tumor tissue in human tumors was less than 0.01% of the injection dose (Björnmalm et al., 2017). Gupta and Sharma. (2021) proposed magnetic dots coated with carboxymethyl-stevioside as a magnetic hyperthermia agent for GBM treatment. These magnetic dots showed significant water stability, and their specific absorption rate was 209.25 W/g under an alternating magnetic field of 359 kHz and 188 Oe. They also induced notable anti-migration and anti-invasive effects on GBM C6 cells by inhibiting the gene expression of matrix metalloproteinases 2 and 9. The key to solving these problems is controlling the amount of magnetic materials that can reach the tumor microenvironment. For this, doping in an appropriate proportion can improve the magnetism of MNPs (Li et al., 2021). Due to the high specific surface area volume ratio, van der Waals force, and strong dipole-dipole interaction, MNPs tend to agglomerate, resulting in increased particle size and reduced magnetism. The high polydispersity of nanoparticles can also reduce the magnetic heating capacity of MNP systems. Therefore, nanoparticle size crucially affects the magnetic and thermal efficiency of nanosystems. In biomedical applications, MNPs with a small diameter (10–100 nm) and narrow size distribution are preferred to prevent their rapid removal from the systemic circulation by the reticuloendothelial system (Cheng et al., 2021). In addition, surface coatings of nanoparticles [such as inorganic materials (alumina or silica), polymers (dextran, chitosan, polyethylene glycol, or stevioside), fatty acids (oleic acid), and liposomes] prevent aggregation and may contribute to colloidal stability through space and electrostatic repulsion (Karimi et al., 2013; Jamari et al., 2020).

Heat shocks can induce the expression of various heat shock proteins (HSPs), which act as molecular chaperones to protect proteins from thermal denaturation, assist protein folding, and induce heat tolerance in cells (Gong et al., 2012). HSP 27, 70, 73, and 90 are considered the key constitutively overexpressed HSPs in GBMs and play an essential role in cancer cell heat resistance against MHCT (Lee Titsworth et al., 2014). Thus, besides improving effective magnetism at the tumor microenvironment, methods targeting these heat shock proteins can also make tumor cells sensitive to magnetic hyperthermia therapy. Possible strategies include specifically inducing an immune response towards these tumor-specific overexpressed HSPs in GBM cells or using HSP gene inhibitors. The heat-induced antitumor immune response is also a new research direction.

#### 2.5.6 Immunotherapy

To date, the standard high-grade GBMs therapy concerns multidisciplinary approaches, containing maximum surgical resection, radiotherapy, and chemotherapy. But the complete resection is nearly impossible because of the invasiveness of GBM, and the recurrence of tumor is practically inevitable even in patients undergoing multimodal therapy. Moreover, these recurrent tumors are often resistant to chemotherapy and radiotherapy (Leiva-Salinas et al., 2017). Thus, it is necessary to develop new therapeutic approaches against GBM. Immunotherapy consists in stimulating the patient’s immune system to make it identify and attack malignant tumors through continuous anti-tumor immunity. However, many obstacles hinder the application of immunotherapy in the clinical practice. The first is the complexity of tumors. Tumors have various immune escape mechanisms. Second, the immune environment of brain is distinct from that in other organs, which is unable to produce an immune response against tumors (Zanganeh et al., 2019). Due to the BBB, the transport of immune effectors from the blood to the brain is limited. Moreover, although activated circulating T lymphocytes are present in the central nervous system, there are few naive T cells (Su et al., 2017).

Another challenge limiting immunotherapy against GBM is that various mechanisms promote immunosuppression inside and around the tumor. GBM is classified as a cold immune tumor, and its microenvironment represents an immune desert with little to no immune effector cell infiltration. Key factors of the glioblastoma-mediated immune cold microenvironment contain the abundance of CD4^+^CD25+FOXP3+ regulatory T cells (Tregs) and myeloid cells, as well as immunosuppressive cytokines and secretory factors produced by tumor cells like transforming growth factor-β, interleukin 6 and interleukin 10 (Reardon et al., 2017). GBM grading is related to Treg infiltration into tumors. In malignant GBM, tumor-resident Tregs express high levels of PD-1 (Lowther et al., 2016), an essential inhibitory receptor expressed in activated T cells which is significant in the immune response (Sharpe and Pauken, 2018). Thus, although there is a small amount of immune cell infiltration, immune cells are often in a low response state due to immunosuppressive signals (Woroniecka et al., 2018).

A primary mediator of immunosuppression in GBM patients is tissue hypoxia, which activates signal transducers and activators of transcription 3 (STAT3) and an immunosuppressive signaling pathway which promotes the production of hypoxia-inducible factor-1-alpha (HIF1A). However, it then induces Treg activation and vascular endothelial growth factor (VEGF) synthesis (Almiron Bonnin et al., 2018).

In the face of the many challenges in immunotherapy, nanomaterials can be used as a breakthrough point. Nanomaterials can deliver drugs to tumors and induce cytotoxic anti-tumor T cell responses. Cancer vaccines with better efficacy can be designed by combining immunomodulators and antigens, direct targeting and T cell functionalization, nucleic acid delivery, adjuvants, immune checkpoint inhibitors, and inhibitory tumor microenvironment regulation (Riley et al., 2019). In a current research, poly (lactic-co-glycolic acid) nanoparticles modified by Angiopep-2 and IP10-EGFRvIIIscFv fusion proteins crossed the BBB and amassed in brain tissue. After binding to cytotoxic T lymphocytes, the nanoparticles notably increased the immune response and antitumor activity in a GBM model (Wang et al., 2018).

To re-adjust the inhibited GBM immune microenvironment, Tong Fei *et al.* designed a nanocomposite drug based on a polyglycerol functionalized doxorubicin-containing nanodiamond. The nanocomposite regulated the immunosuppressive microenvironment of GBM by activating autophagy, thereby stimulating the immune response. This rebuilding promoted the anti-glioblastoma immune response and strengthened the activation of dendritic cells (Li et al., 2019).

The tumor vascular laminin-411 (α4β1γ1) is associated with the high expression of tumor stem cell markers (Notch, CD133, Nestin, c-Myc) and a shorter survival time for GBM patients. Tao *et al.* designed a nano-bioconjugate which is able to cross the BBB to inhibit laminin-411. This nanobioconjugate targeting the tumor microenvironment prolonged animal survival and inhibited cancer stem cell markers in mice carrying intracranial GBM (Sun et al., 2019).

The low accumulation of antigens in antigen-presenting cells is another obstacle to effective immunotherapy against brain tumors. This is related to the low activation of antigen-presenting cells in GBM. Two approaches could solve this problem: enhancing the antigen-loading capacity of nanovaccines or adding substances that can sensitize antigen-presenting cells to nanomedicines. A hybrid “cluster bomb” nanovaccine, based on zinc oxide and triblock-copolymer nanoparticles, stimulated cellular and humoral immunity and increased the survival time of tumor-bearing mice (Shen et al., 2019). Another study combined exosomes derived from GBM with α-galactosylceramide (natural killer T-cell activator). Used subcutaneously in glioblastoma-bearing rats, it increased interferon γ and tumor necrosis factor α production and promoted the immune response (Liu H. et al., 2017).

#### 2.5.7 Summary

**FIGURE 2:**
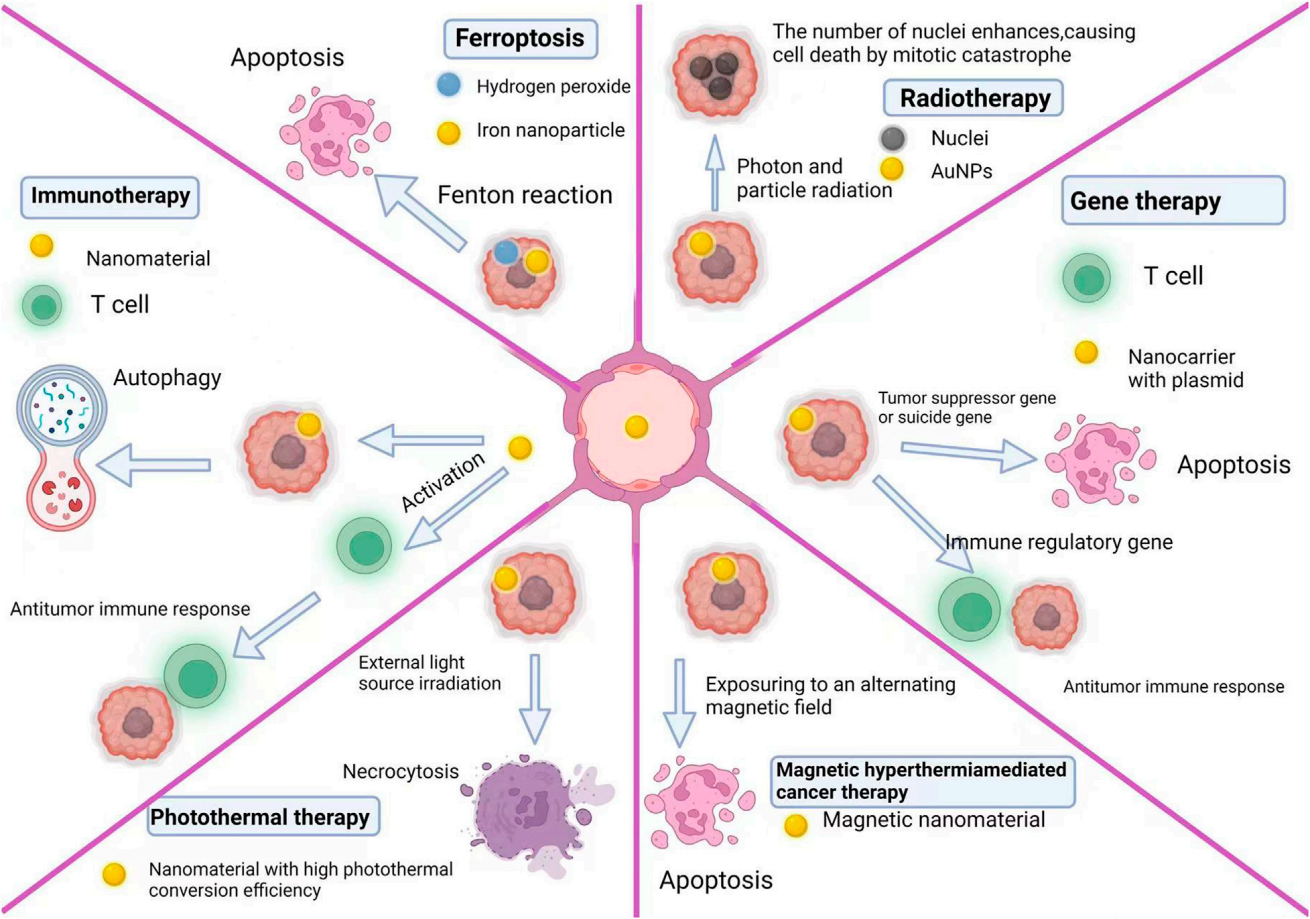
Glioblastoma therapy mechanism of nanomaterials.

Because of the heterogeneity, the complexity of the tumor microenvironment, the multiple immune escape mechanisms, and the weak sensitivity of the brain immune system, handling GBM from a single dimension is limited. GBM treatments need to be multimodal. However, combining different methods for killing glioma cells is insufficient. Comprehensive glioblastoma treatments should also target the tumor microenvironment, and stimulate immune cells of the brain (Figure 2).

## 3 Summary and outlook

This article reviews the definition, classification, diagnosis, and therapeutic applications of nanomaterial-based GBM treatments. Currently, the clinical application of nanoparticles and the complexity of GBM itself still face numerous challenges. Designing nanomaterials more suitable for clinical applications require optimizing nanomaterials by understanding the transport regulation mechanism of the BBB, the composition of the GBM tumor microenvironment and its influence on the BBB. Carefully studying the mechanisms of action of nanomaterials on the brain, discovering new properties of nanomaterials, improving their synthesis, and exploring new and promising drug delivery systems are crucial for developing clinical applications of nanomaterials in the diagnosis and treatment of glioblastoma. Most importantly, since single-dimension diagnostic and treatment methods are limited, future studies should focus on multi-dimensional nanomaterials. With further research, the clinical nano-diagnosis and treatment system for GBM is expected to improved.
